# Measuring adequacy of the midwifery workforce using standards of competency and scope of work: Exploring the density and distribution of midwives in three low- and middle-income countries using cross-sectional and geospatial data

**DOI:** 10.1371/journal.pone.0284034

**Published:** 2023-04-06

**Authors:** Jewel Gausman, Sowmya Ramesh, Richard M. Adanu, Delia A. B. Bandoh, Jeff Blossom, Suchandrima Chakraborty, Ernest Kenu, Nizamuddin Khan, Ana Langer, Magdalene A. Odikro, Niranjan Saggurti, R. Rima Jolivet

**Affiliations:** 1 Women and Health Initiative, Department of Global Health and Population, Harvard University T.H. Chan School of Public Health, Boston, Massachusetts, United States of America; 2 Population Council, New Delhi, India; 3 Department of Population, Family, and Reproductive Health, University of Ghana School of Public Health, Accra, Ghana; 4 Department of Epidemiology and Disease Control, University of Ghana School of Public Health, Accra, Greater Accra, Ghana; 5 Center for Geographic Analysis, Harvard University, Cambridge, Massachusetts, United States of America; Jhpiego, UNITED STATES

## Abstract

**Background:**

A global midwifery shortage hampers the goal of ending preventable maternal/newborn mortality and stillbirths. Whether current measures of midwifery workforce adequacy are valid is unknown. We compare two measures of density and distribution of midwifery professionals to assess their consistency, and explore how incorporating midwifery scope, competency, and the adjusting reference population impacts this critical metric.

**Methods and findings:**

We collected a census of midwives employed in eligible facilities in our study settings, (422 in Ghana; 909 in India), assessed the number practicing within the scope of work for midwifery professionals defined in the International Labor Organization International Standard Classification of Occupations, and whether they reported possessing the ICM essential competencies for basic midwifery practice. We altered the numerator, iteratively narrowing it from a simple count to include data on scope of practice and competency and reported changes in value. We altered the denominator by calculating the number of midwives per 10,000 total population, women of reproductive age, pregnancies, and births and explored variation in the indicator. Across four districts in Ghana, density of midwives decreased from 8.59/10,000 total population when counting midwives from facility staffing rosters to 1.30/10,000 total population when including only fully competent midwives by the ICM standard. In India, no midwives met the standard, thus the midwifery density of 1.37/10,000 total population from staffing rosters reduced to 0.00 considering competency. Changing the denominator to births vastly altered subnational measures, ranging from ~1700% change in Tolon to ~8700% in Thiruvallur.

**Conclusion:**

Our study shows that varying underlying parameters significantly affects the value of the estimate. Factoring in competency greatly impacts the effective coverage of midwifery professionals. Disproportionate differences were noted when need was estimated based on total population versus births. Future research should compare various estimates of midwifery density to health system process and outcome measures.

## Introduction

An effective midwifery workforce is critical for ending preventable maternal/newborn mortality and stillbirths. Quality maternal newborn care across the continuum of care from pre-pregnancy through antenatal stages [1–3], intrapartum care and skilled birth attendance [4–6], and postpartum care for women and infants [7–10] can reduce mortality and improve pregnancy outcomes [11]. Recent estimates indicate that midwifery professionals can effectively delivery 59–90% of essential maternal newborn care interventions [12, 13], especially when educated and regulated to global standards [14]. Moreover, with universal coverage of such interventions, high-quality midwifery care could avert 4.3 million preventable deaths annually by 2035, representing approximately two-thirds of all maternal and neonatal deaths and stillbirths during that period [15]. This goal, however, may be hampered by the well-documented global shortage of qualified midwives [13, 16–19].

Achieving an adequate midwifery workforce is essential to advance a high-functioning maternity care system. “Adequacy” includes dimensions of quantity and quality—a sufficient number of midwifery professionals will only be adequate to meet the needs of the population if they possess the required skills and are able to exercise those skills. Further, their distribution across geography and throughout the health system is an important component of workforce adequacy. Ensuring robust monitoring of the density and distribution of the midwifery workforce has significant implications for supporting equitable access to essential maternal and newborn health (MNH) care. There is more than one approach to measure the adequacy of the midwifery workforce, and the validity of currently available measures is unknown.

In 2015, the World Health Organization (WHO) released “Strategies toward Ending Preventable Maternal Mortality (EPMM)”, a report outlining 11 key themes for reducing maternal mortality during the Sustainable Development Goal (SDG) period [20]. Subsequently, a panel of stakeholders selected indicators for a monitoring framework for the EPMM strategies, using a broad scoping exercise to map candidate indicators for the 11 key themes from existing measures. More than 150 individuals from 78 organizations evaluated the candidate indicators against pre-determined selection criteria including relevance to the theme, importance, and utility [21]. The stakeholders selected two slightly different indicators to monitor the density and distribution of the midwifery workforce (Box 1).

**Box 1. Indicator metadata.**

**Table:** 

**Indicator 12: Health worker density and distribution (per 1,000 population)** Disaggregation allows for identification of density and distribution of midwifery professionals (classified by ILO job tasks)			
Numerator Number of health workers by cadre	Denominator Total population	Disaggregator(s) **Cadre**: General and specialist practitioners, nursing and midwifery professionals, traditional and complementary medicine professionals; **Distribution**: Place of employment (urban/rural), Subnational (district)	Data Source Health worker registry
Reference: SDG indicator metadata, Indicator 3.c.1., 2022. UN Stats https://www.who.int/activities/improving-health-workforce-data-and-evidence			
**Indicator 13: Density of midwives, by district (by births)** This indicator tracks the number of midwives per 1,000 births			
Numerator Number of midwives in a district	Denominator All births/pregnancies in a district	Disaggregator(s) None	Data Source Surveys
Reference: World Health Organization, 2014. Consultation on improving measurement of the quality of maternal, newborn and child care in health facilities.

The first, “Health worker density and distribution (per 10,000 population),” (SDG target 3.c.1), is defined as the number of health workers per 10,000 population, which is disaggregated by occupation including midwifery professionals [22]. The second, “Density of midwives, by district (by births),” defined as the number of midwives by district per 1000 births in the district, is an alternative measure of the same construct [23]. Stakeholders prioritized the first indicator for its potential to drive improvements in health system strengthening to meet the needs of women and girls. It was prioritized, specifically to address priority recommendations related to national workforce management and regulatory support by national governments to deploy midwives in sufficient numbers to ensure universal coverage of all essential services that fall within the midwifery scope of practice. In that context, the adequacy of the midwifery workforce was prioritized within the context of a total MNH health workforce that also includes other skilled maternity care providers in adequate numbers to meet population needs. The selection of the second measure reflects its relevance to reduce inequities in access to essential maternal health care and to refine and better specify the population in need. While the indicators seek to measure the same phenomenon (coverage), these two estimates of density and distribution of midwifery personnel use different parameters for both numerator and denominator (see Box 1).

Coverage is defined as the proportion of individuals receiving (or in this case able to provide) “an intervention or practice (numerator) among the population in need of that intervention or practice (denominator)” [24]. To our knowledge, there is no accepted reference standard for optimal density of the midwifery workforce for population coverage. Questions remain about the optimal denominator to assess adequate midwifery workforce coverage. For example, coverage indicators that employ population-based denominators using total population estimates may not accurately reflect the need for interventions that apply to a specific segment of the population [25]. Thus, the current definition of density per SDG 3.c.1 does not account for the precise population in need of midwifery care. Changes in the denominator may affect the value of the estimate, although there is no agreement on the best denominator to capture adequate coverage of the midwifery workforce.

Similarly, there are several challenges in estimating the numerator for this construct for measurement. First, which health workers should be counted in the numerator? Not all countries have an independent cadre of midwives, and it may be difficult to distinguish among nurses, midwives, and other related health workers in many settings [26], especially as trained midwives may not be working in that capacity within the health system [27]. Limiting the discussion of who should be counted in the midwifery workforce to intrapartum care alone, a review cited in the most recent State of the World’s Midwifery Report (2021) identified 102 unique names used in low- and middle-income countries to describe health workers who attend births, and not all countries adhere to the 2018 definition of a skilled health personnel providing care during childbirth [17, 28, 29].

The International Confederation of Midwives (ICM) indicates that a person meeting the ICM definition of a midwife [30], educated to the ICM Global Standards for Midwifery Education [31], and practicing to the level of the ICM Competencies [32] is considered a midwife and can use the title, regardless of their official credential or job title. Similarly, the International Labour Organization (ILO) notes that distinctions between nursing and midwifery professionals should be made on the basis of the nature of the work they perform (scope of practice), which is captured in the tasks specified in the ILO Classification of Occupations (2012) ISCO-8 [33] (Box 2). That document further specifies that neither the training nor qualifications held by individuals or that are predominant in the country are the main factor in making this distinction between nursing and midwifery professionals, which varies greatly between countries. However, this lack of specificity hampers efforts to define the numerator for this indicator with precision.

**Box 2. Global standard definitions for midwives and midwifery professionals.**

**Table:** 

*ICM Definition of a Midwife*	*ILO Scope of Practice for Midwifery Professionals*
A midwife is a person who has successfully completed a midwifery education programme that is based on the ICM Essential Competencies for Midwifery Practice and the framework of the ICM Global Standards for Midwifery Education and is recognized in the country where it is located; who has acquired the requisite qualifications to be registered and/or legally licensed to practice midwifery and use the title ‘midwife’; and who demonstrates competency in the practice of midwifery.	Midwifery professionals plan, manage, provide and evaluate midwifery care services before, during and after pregnancy and childbirth. They provide delivery care for reducing health risks to women and newborn children, working autonomously or in teams with other health care providers. Midwifery associate professionals provide basic health care and advice before, during and after pregnancy and childbirth. They implement care, treatment and referral plans usually established by medical, midwifery and other health professionals.

*ADD REFERENCES; LABEL, plos medicine author guidelines

Moreover, coverage indicators are criticized because they do not reflect information on quality or content of care provided [34]. To produce a valid estimate of the number (density) of midwifery professionals needed for adequate coverage of the population, a necessary assumption is that all midwifery professionals can provide the same intervention (scope of practice) to the same degree of quality (competency). The ILO Classification of Occupations outlines a standard global framework for the scope of practice of midwives and midwifery professionals; ICM provides a standard definition of a midwife and promotes a global standard for midwifery core competencies, including knowledge, skills, and behaviors [30, 32]. In reality, midwifery scopes of practice and competencies vary across settings, posing threats to this assumption. Neither indicator accounts for a midwife’s competencies to deliver essential services, nor for aspects of the enabling environment that allow them to effectively exercise those competencies. It is thus challenging to produce a valid estimate of the number of midwives required to provide essential services to the population in need without accounting for midwives’ scope of practice, skills/competencies, and the enabling environment, which must be factored in to calculate a measure of “effective coverage” [35–37] by the midwifery workforce.

Many guiding organizations suggest frameworks for strengthening the midwifery workforce to meet the shortfall [38–42]. For example, the ICM’s Midwifery Services Framework [43] provides guidance to countries through a four-stage methodology that was initiated in eight low- and middle-income countries by 2018. This framework guides countries to determine the minimum service package of essential MNH care, to plan the organization of care services for the package, and the necessary number and distribution of midwives to deliver them. The effectiveness of such midwifery workforce strengthening guidelines and activities relies on the availability of valid measures for the density and distribution of midwives, including validity of the underlying constructs that constitute the numerator and denominator of this critical health system strengthening indicator.

This study seeks to provide essential information to support midwifery workforce strengthening by comparing estimates from two measures of the density and distribution of midwives and midwifery professionals to assess their convergent validity, or whether they are consistent and track reliably with each other. We calculate their value using a range of numerators and denominators derived from primary data sources as well as population censuses and estimates, accounting for variations in competency of the midwifery workforce in calculating the numerator as well as the relevance of different subpopulations in the denominator to the construct for measurement. We report variation in the values of the indicator using different parameters The purpose of this research is to provide data to explore whether indicator adjustment would give a better estimate of this construct and to suggest potential solutions to better operationalize measures of the midwifery workforce.

## Methods

### Study setting

The country research settings for this study were chosen as part of a larger research project, in which low- or middle-income countries with significant maternal mortality burden were considered and settings were purposively selected based on geographic diversity and research partner capacity. In each country, four subnational study settings were selected based on a composite index of key maternal health indicators to reflect varying levels of health system performance. We selected a state or region from the top quartile and one from the bottom quartile, and within those selected a district/province from the top and bottom quartiles based on the index. This study took place in four subnational geographic areas in Ghana (Bunkpurugu Yunyoo and Tolon in the Northern Regions, and Techiman and Sunyani Municipal in the Brong-Ahafo Region) and India (Gonda and Meerut in the state of Uttar Pradesh, and Krishnagiri and Thiruvallur in the state of Tamil Nadu). More details on the selection of study settings are previously published [44].

### Ethical considerations

The Institutional Review Board (IRB) of the Harvard T.H. Chan School of Public Health approved this study on 4 September 2019. The research is classified, using Harvard’s Data Security Policy, as Level 4 Data. The study also was approved in Ghana by the Ghana Health Service Ethical Review Board (approval ID: GHS-ERC022/08/19); and in India by the national population council IRB (approval ID: 889) and local Sigma-IRB (approval ID: 10052/IRB/19-20).

## Primary data collection

We collected primary data on a census of midwives to calculate the number of facility-based midwives practicing in each study setting and to determine the extent to which the midwifery workforce reflected the ICM essential competencies for midwifery practice. In Ghana, cross-sectional data were collected from midwives employed at all public and private-registered facilities that provide maternal health-related services in each study area, resulting in 13 tertiary-level facilities, 28 secondary-level facilities, and 74 primary-level facilities being included in the study. In India, all secondary and tertiary facilities offering maternal health-related services were included in the study; however, due to the large number of primary-level health facilities that fit the study’s definition, a random sample of 20 facilities at this level were selected for inclusion from each study area. In total, 20 tertiary-level facilities, 54 secondary-level facilities, and 84 primary-level facilities were included in the study.

We determined eligibility for this study using the ILO classification of occupations based on scope of work and accepted all participants who met the definition for either midwifery professional or midwifery associate professional, since both occupations provide direct health care services before, during, and after pregnancy and childbirth. This definition was pragmatic, given the lack of an independent cadre of health professionals designated as midwives in India, and based on the objective of the indicators under study, which seek to calculate the adequacy of the midwifery workforce according to global standards in each setting. All midwives working in eligible facilities who met our study definition were identified and recruited through a multi-step process. In the first step, unit chiefs in each facility were asked to provide a list of all healthcare workers whose scopes of work reflected ILO ISCO-08 occupational classification for nursing and midwifery professionals (MPs) and midwifery associate professionals (MAPs) [33], irrespective of the healthcare worker’s official or working title. ILO defines MPs as employees who “plan, manage, provide, and evaluate midwifery care services before, during, and after pregnancy and childbirth…provide delivery care for reducing health risks to women and newborn children, working autonomously or in teams with other health care providers,” and MAPs as employees who “provide basic care and advice before, during, and after pregnancy and childbirth…implement care, treatment, and referral plans usually established by medical, midwifery, and other health professionals.” In the second step, healthcare workers identified as MPs or MAPs in each facility were contacted and individually screened for eligibility by our trained field research teams (in person in Ghana and by telephone in India) to verify that their scope of work matched the ILO definition based on the types of tasks that they performed. As a final step in the recruitment process, we invited all MPs and MAPs who confirmed that their scopes of work aligned with the ILO classification to participate in the study. In facilities employing ≤50 midwives, we invited all midwives to participate. In facilities employing >50 midwives, we invited a random sample of 50 midwives from the verified list to participate. Participants were eligible if they were working as healthcare providers in a capacity that met the ILO definitions for MPs or MAPs, were old enough to provide consent (at least 18), and proficient in the local language or English. All participants provided informed consent prior to participating in the study.

Eligible midwives were asked to complete a task survey based on the ICM “Essential Competencies for Basic Midwifery Practice” [32]. ICM competencies are organized within a framework that reflects four broad categories: 1) general competencies, 2) pre-pregnancy and antenatal care, 3) care during labor and birth, and 4) ongoing care of women and newborns. Within each category are competencies identified by ICM that represent the expected outcomes of midwifery pre-service education and are linked to evidence of clinical effectiveness [45]. Competencies that fall within each category are accompanied by a list of indicators that represent the minimum set of knowledge, skills, and behaviors required to perform the competency to a safe standard of practice in any setting. In the survey, midwives were asked to report whether they had the necessary skills to perform the behaviors associated with each ICM competency identified in Categories 2, 3, and 4. We omitted Category 1 from the survey because the associated skills and behaviors were considered too general and abstract to be easily assessed.

The task survey was administered as a verbal structured interview in Ghana, or via telephone in India. The survey asked respondents to self-report whether they had “none,” “some,” “most,” or “all” of the necessary skills to perform each behavior identified for each ICM competency in Categories 2, 3, and 4. In total, the task survey included 112 questions on specific behaviors. In total, 98.8% (413/418) of eligible midwives in Ghana, and 98.8% (766/775) of eligible midwives in India completed the task survey.

### Population data

We estimated the total population, number of women of reproductive age, number of pregnancies, and number of births using ArcGIS Pro software, version 2.6 [46] in each study setting. The GIS shapefile was used to define the administrative boundaries of each study setting, which we obtained from the Database of Global Administrative Areas, version 2.0 [www.gadm.org]. With these administrative boundaries, we used the open-access GIS dataset *WorldPop*, [www.worldpop.org] which uses census data, United Nations population estimates, and satellite imagery [47] to estimate the human population within the administrative boundaries identified for each setting. Total population estimates were available from the year 2020 and at a resolution of 100 m. Estimates of pregnancies and births were available only for the year 2015 and at a resolution of 1 km. The ArcGIS resample function with the Nearest option was used to increase resolutions of the pregnancies and births rasters to 100 m to match the resolution of the total population raster. The top-down population modeling method was used to identify rural areas, small settlements, and uninhabited areas [47, 48].

### Analysis

We defined the numerator by beginning with the broadest definition and iteratively narrowing the definition to include data on scope of practice and competency. To obtain the broadest estimate, we aggregated the number of midwives extracted from facility records in each study area. We then narrowed the definition by removing from the list those midwives whose scopes of work did not meet the ILO definition for MP or MAP after individual screening. Finally, we integrated data on competency derived from the task survey to further narrow the definition of the numerator. Within each ICM competency, we assigned a value of 0, 1, 2, or 3 to each behavior corresponding to whether a midwife indicated having “none,” “some,” “most,” or “all” of the skills necessary to perform the behavior. We calculated the mean score reported across the behaviors associated with each competency. We then calculated a mean score of all competencies within each of the three categories. We calculated the overall mean score by taking the average score of all three categories. The intention of this approach was to ascribe equal importance to each category of care regardless of the number of behaviors and competencies associated with each category. Midwives who had a mean score of ≥2 were considered to have “most” of the skills necessary to reflect the ICM competencies, while midwives with a score of 3 were considered to have “all” of the necessary skills. We descriptively assessed the competency of the midwifery workforce examining the proportion of midwives across study settings who were considered “mostly” or “fully” competent based on their score and used chi-squared tests to examine whether there were systematic differences in competency based on location of the facility in which the midwife worked (rural versus urban) or level of health facility (primary, secondary, or tertiary).

Missing responses to individual survey questions were assumed to be missing at random. The largest number of missing responses to any given question was 18 in Ghana (4.35% of respondents) and three in India (0.004% of respondents). Respondents who had missing responses to >10% of the 112 behavioral questions on the survey were excluded from the analysis. This step resulted in 0.01% (n = 4) of the sample being excluded in Ghana but none in India.

Using these data, we critically examined different approaches to operationalizing the indicator by varying the numerator and denominator. To examine the numerator, we calculated the number of midwives in the study areas who were: 1) extracted from facility records, 2) screened and determined to have scopes of practice that fit the ILO definition of MP or MAP, and 3) “mostly” or “fully” competent. We applied each of these numerators to calculate the value of the indicator representing the density of midwives per 10,000 total population in the study areas. To examine the denominator, we iteratively varied the denominator using population data obtained from GIS estimates. We calculated the number of midwives in the study areas (as extracted from facility records) per 10,000 total population, women of reproductive age, pregnancies, and births and descriptively explored variation in the indicator values.

## Results

### Numerator

Table 1 provides the number of midwives identified from facility records and those retained after individual screening. In Ghana, 422 midwives were identified from facility records, ranging from 40 in Bunkpurugu Yunyoo to 217 in Sunyani. The majority of midwives worked in urban areas (45%, n = 229) and at primary health centers (58%, n = 245). After individual screening, two health workers identified as midwives from facility records did not have scopes of work consistent with the ILO occupational classification for MPs and MAPs and were removed from the study. In India, 909 health workers were identified from facility records; however, individual screening found that only 775 had scopes of work that reflected the ILO occupational classification for MPs and MAPs (15% reduction). The number of health workers removed from the counts varied by district—no midwives were removed after screening in Gonda (n = 160) and Meerut (n = 173), while nearly 30% of midwives were removed after screening in Krishnagiri (n = 96) and 15% in Thiruvallur (n = 38). The majority of midwives who were removed after screening were located in rural areas (18%, n = 92) and worked in primary-level facilities (19%, n = 113).

**Table 1:** Health workers identified from facility records.

	# of Midwifes from Staffing Roster	# of Midwives who fit ILO Definition after Screening	% Difference between Roster and Verified through Screening
**Ghana**			
*Study Area*			
Bunkpurugu Yunyoo	40	40	0.00%
Tolon	104	104	0.00%
Sunyani Municipal	217	215	0.92%
Techiman	61	61	0.00%
*Location*			
Urban	229	227	0.87%
Rural	191	191	0.00%
*Facility Level*			
Tertiary	62	62	0.00%
Secondary	115	115	0.00%
Primary	245	243	0.82%
*Total*	422	420	0.47%
**India**			
*Study Area*			
Gonda	160	160	0.00%
Krishnagiri	330	234	29.09%
Meerut	173	173	0.00%
Thiruvallur	246	208	15.45%
*Location*			
Urban	385	363	5.71%
Rural	504	412	18.25%
*Facility Level*			
Tertiary	99	99	0.00%
Secondary	188	187	0.53%
Primary	602	489	18.77%
*Total*	909	775	14.74%

The competency of the midwifery workforce varied between countries. Table 2 presents the percentage of midwives who had “none,” “some,” “most,” or “all” of the skills related to each competency, category, and overall. Most notably, no MPs or MAPs met the ICM minimum standard for competency in India, while 14% (n = 59) possessed all necessary skills to perform all behaviors associated with included ICM competencies in Ghana. Relaxing the definition of competency to include midwives who had “most” or “all” of the skills resulted in a similar percentage of competent midwives between countries: 76% (n = 312) in Ghana and 82% (n = 628) in India. While Ghana had the highest number of midwives who indicated they possessed all the skills to meet all ICM competencies, the country also had the largest percentage of midwives reporting that they possessed none of the skills. Nearly 8% (n = 32) of midwives surveyed in Ghana possessed none of the skills overall, compared to none in India.

**Table 2:** Proportion of midwives reporting having all, most, some, or none of the skills to perform the behaviors associated with ICM competencies in Ghana and India.

	Ghana (n = 414)				India			
	None	Some	Most	All	None	Some	Most	All
Category 2: Provide Pre-pregnancy and Antenatal Care	29 (7.06)	65 (15.82)	238 (57.91)	79 (19.22)	0 (0.00)	172 (22.45)	594 (77.55)	0 (0.00)
2.a. Provide pre-pregnancy and antenatal care	18 (4.38)	68 (16.55)	176 (42.82)	149 (36.25)	0 (0.00)	101 (13.19)	578 (75.46)	87 (11.36)
2.b. Determine health status of women	16 (3.89)	64 (15.57)	146 (35.52)	185 (45.01)	0 (0.00)	135 (17.62)	508 (66.32)	123 (16.06)
2.c. Assess fetal well-being	49 (11.92)	56 (13.63)	93 (22.63)	213 (51.82)	0 (0.00)	164 (21.41)	509 (66.45)	93 (12.14)
2.d. Monitor the progression of pregnancy	29 (7.06)	52 (12.65)	86 (20.92)	244 (59.37)	0 (0.00)	83 (10.84)	563 (73.50)	120 (15.67)
2.e. Promote and support health behaviors that improve well being	22 (5.35)	49 (11.92)	79 (19.22)	261 (63.50)	0 (0.00)	76 (9.92)	588 (76.76)	102 (13.32)
2.f. Provide anticipatory guidance related to pregnancy, birth, breastfeeding, parenthood, and change in the family	24 (5.85)	51 (12.44)	88 (21.46)	247 (60.24)	0 (0.00)	159 (20.76)	500 (65.27)	107 (13.97)
2.g. Detect, stabilize, manage, and refer women with complicated pregnancies	58 (14.15)	67 (16.34)	82 (20.00)	203 (49.51)	7 (0.91)	219 (28.59)	477 (62.27)	63 (8.22)
2.h. Assist the woman and her family to plan for an appropriate place of birth	28 (6.81)	63 (15.33)	68 (16.55)	252 (61.31)	0 (0.00)	75 (9.79)	569 (74.28)	122 (15.93)
2.i. Provide care to women with unintended or mistimed pregnancy	60 (14.60)	67 (16.30)	86 (20.92)	198 (48.18)	34 (4.44)	211 (27.55)	464 (60.57)	57 (7.44)
Category 3: Care During Labor and Birth	64 (15.57)	63 (15.33)	153 (37.23)	131 (31.87)	0 (0.00)	166 (21.67)	597 (77.94)	3 (0.39)
3.a. Promote physiologic labor and birth	64 (15.57)	50 (12.17)	123 (29.93)	174 (42.34)	1 (0.13)	218 (28.46)	511 (66.71)	36 (4.70)
3.b. Manage a safe spontaneous vaginal birth and prevent complications	79 (19.22)	48 (11.68)	117 (28.47)	167 (40.63)	5 (0.65)	155 (20.23)	570 (74.41)	36 (4.70)
3.c. Provide care of the newborn immediately after birth	50 (12.17)	58 (14.11)	83 (20.19)	220 (53.53)	1 (0.13)	102 (13.32)	424 (55.35)	239 (31.20)
Category 4: Ongoing Care of Women and Newborns	13 (3.16)	63 (15.33)	160 (38.93)	175 (42.58)	1 (0.13)	92 (12.01)	639 (83.42)	35 (4.57)
4.a. Provide postnatal care for the healthy woman	29 (7.07)	62 (15.12)	88 (21.46)	231 (56.34)	0 (0.00)	88 (11.49)	527 (68.80)	151 (19.71)
4.b. Provide care to healthy newborn infant	29 (7.07)	62 (15.12)	88 (21.46)	231 (56.34)	0 (0.00)	61 (7.96)	504 (65.80)	201 (26.24)
4.c. Promote and support breastfeeding	8 (1.95)	30 (7.32)	71 (17.32)	301 (73.41)	0 (0.00)	37 (4.83)	445 (58.09)	284 (37.08)
4.d. Detect, treat, and stabilize postnatal complications in woman and refer as necessary	36 (8.78)	54 (13.17)	77 (18.78)	243 (59.27)	0 (0.00)	102 (13.32)	528 (68.93)	136 (17.75)
4.e. Detect, stabilize, and manage health problems in newborn infant and refer if necessary	35 (8.75)	42 (10.50)	85 (21.25)	238 (59.50)	0 (0.00)	112 (14.62)	473 (61.75)	181 (23.63)
4.f. Provide family planning services	11 (2.68)	40 (9.73)	82 (19.95)	278 (67.64)	0 (0.00)	68 (8.88)	381 (49.74)	317 (41.38)
Overall	32 (7.79)	67 (16.30)	253 (61.56)	59 (14.36)	0 (0.00)	138 (18.02)	628 (81.98)	0 (0.00)

Midwives working in rural areas and at primary-level facilities tended to report lower competency than those in urban areas in both countries. In India, 71% (n = 293) of midwives working in rural versus 94% (n = 345) of midwives working in urban areas reported having “most” skills (p<0.001), while 75% (n = 451) of midwives working at the primary level compared to 95% (n = 178) and 92% (n = 89) working at the secondary and tertiary levels, respectively, in India reported having “most” skills (p<0.001). In Ghana, similar patterns were observed by location and level of care. In rural areas, 70% (n = 134) of midwives reported possessing “most” or “all” skills related to ICM competencies, while 82% (n = 186) reported having “most” or “all” skills in urban areas (p<0.01). Further, only 70% (n = 170) of midwives working at the primary level, compared to 80% (n = 92) at the secondary and 92% (n = 57) at the tertiary level, reported possessing “most” or “all” skills (p<0.001).

**Fig 1:**
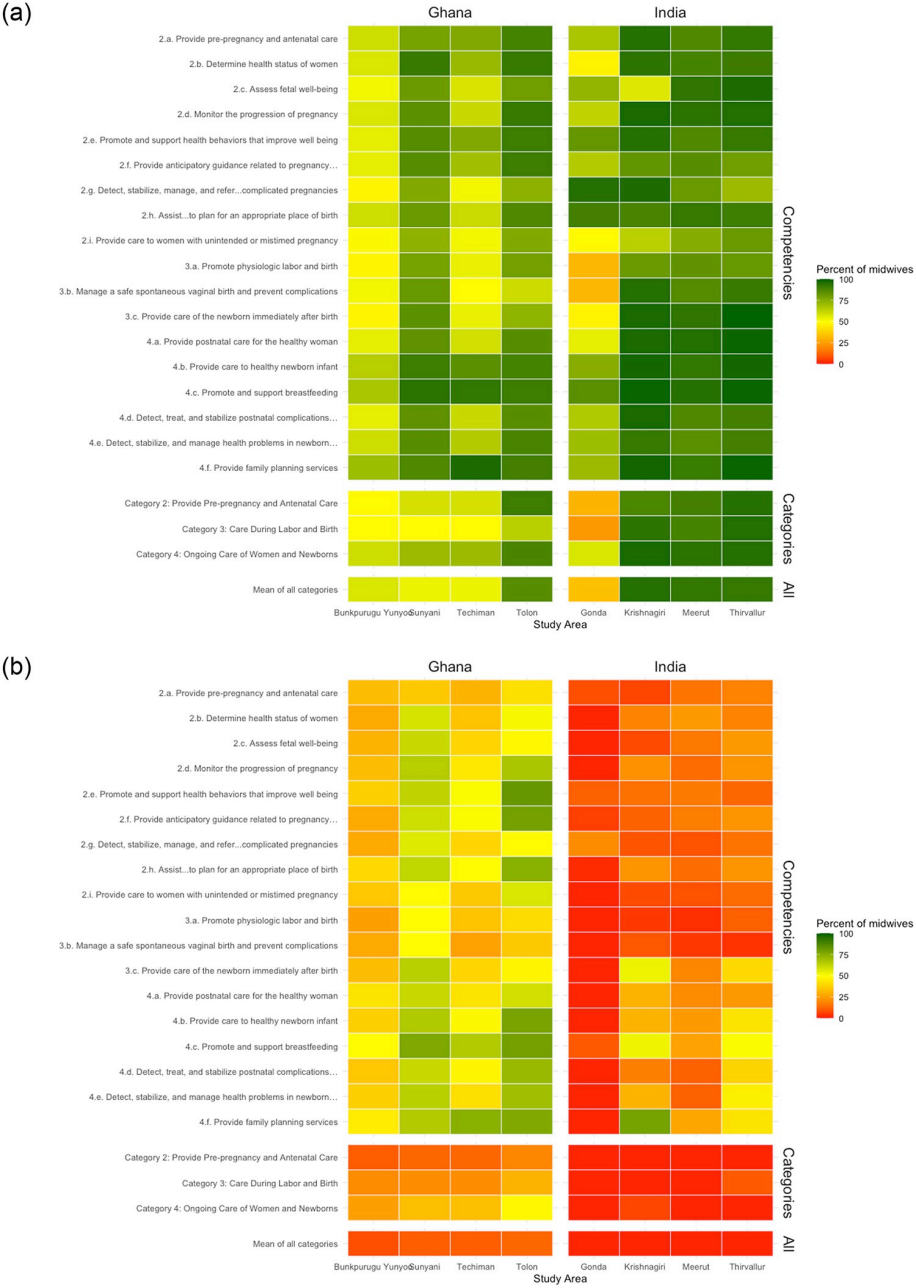
a.: Distribution of mostly competent midwives across study settings according to ICM competency, category, and overall. b.: Distribution of fully competent midwives across study settings according to ICM competency, category, and overall.

The data also illustrate substantial variation in midwife competency across study areas. Fig 1a shows the percentage of midwives in each study area who possessed “most or all” of the skills associated with ICM competencies, while Fig 1b shows the percentage of midwives who possess “all” of the skills associated with the ICM competencies. In India, midwives working in the Gonda district had lowest overall competency scores, with results suggesting that only 34% (n = 54) of midwives were mostly competent, compared to >90% in other districts (95%, n = 222 in Krishnagiri, 93%, n = 161 in Meerut, and 92%, n = 191 in Thiravullur). Overall, the percentage of midwives who were fully competent across all ICM competencies in Ghana ranged from 8% (n = 3) in Bunkpurugu Yunyoo to 18% (n = 39) in Sunyani. In both Ghana and India, competency related to Category 2 was the lowest overall.

### Denominator

Population parameters estimated using GIS data are presented in Table 3 for each study area. The proportion of women of reproductive age within the total population in each study area varied considerably, from approximately 24% in Gonda to 29% in Thiruvallur in India and from approximately 20% in Bunkpurugu Yunyoo to 26% in Tolon in Ghana. Further, the proportion of pregnancies estimated among women of reproductive age varied tremendously across study settings, ranging in India from 6% in Thiruvallur to 15% in Gonda and in Ghana from 15% in Sunyani to 31% in Techiman. Further, the percentage of pregnancies resulting in births was inconsistent across study areas, ranging from 65% in Meerut in India to approximately 75% across all four districts in Ghana.

**Table 3:** Population estimates in subnational geographic areas of India and Ghana.

Study Setting	Total Population	Number of Women of Reproductive Age (WRA)	Percent of WRA in Total Population	Number of Pregnancies	Percent of WRA with Pregnancies	Number of Births	Percentage of Pregnancies Resulting in Birth
**India**	12,456,299	3,341,277	26.82%	347,262	10.39%	232,811	67.04%
Krishnagiri	1,979,808	563,185	28.45%	46,098	8.19%	31,353	68.01%
Thiruvallur	3,896,148	1,124,671	28.87%	65,416	5.82%	44,491	68.01%
Gonda	3,388,142	825,707	24.37%	122,150	14.79%	83,078	68.01%
Meerut	3,192,201	827,714	25.93%	113,598	13.72%	73,889	65.04%
**Ghana**	444,731	97,607	21.95%	21,937	22.47%	16,405	74.78%
Bunkpurugu Yunyoo	186,718	38,269	20.50%	8,494	22.20%	6,352	74.78%
Tolon	52,040	13,391	25.73%	3,880	28.97%	2,901	74.77%
Sunyani	135,426	28,409	20.98%	4,201	14.79%	3,142	74.79%
Techiman	70,548	17,539	24.86%	5,362	30.57%	4,010	74.79%

### Indicator calculations

Alternative calculations of the indicator in which we varied the numerator and denominator are presented in Table 4. In all countries, the value of the indicator decreased considerably as the definition of a midwife shifted from broad (number of midwives obtained from facility records) to narrow (number of midwives who reportedly possessed all skills to perform behaviors associated with ICM competencies). In Ghana, we estimated that there were 8.59 midwives per 10,000 total population when extracting the numerator from facility records; however, this decreased to 1.30 per 10,000 total population when including in the numerator only midwives who reported they were fully competent. In India, no midwives were reported to be fully competent, thus the value of the indicator at its most narrow definition was zero, and there was <1 mostly competent midwife per 10,000 population in all study areas except for Krishnagiri, where there was 1.51.

**Table 4:** Indicator calculations for density and distribution of midwives per 10,000 women of reproductive age, pregnancies, and births.

Study Setting	Number of Midwives (Obtained from Facility Records) per 10,000 Total Population	Redefining the Numerator*			Redefining the Denominator**		
		After Screening for ILO Definition	Meeting Most/All ICM Competencies	Meeting All ICM Competencies	Per 10,000 Women of Reproductive Age	Per 10,000 Pregnancies	Per 10,000 Births
**Ghana**	8.59	8.54	6.99	1.30	39.14	174.14	232.86
Bunkpurugu Yunyoo	2.14	2.14	1.23	0.16	10.45	47.09	62.97
Tolon	19.98	19.98	16.53	2.31	77.66	268.04	358.50
Sunyani	16.02	15.88	12.63	2.73	76.38	516.54	690.64
Techiman	8.65	8.65	4.39	0.85	34.78	113.76	152.12
**India**	1.37	1.13	0.92	0.00	5.1	49.83	73.26
Gonda	1.30	1.30	0.44	0.00	5.35	36.18	53.2
Krishnagiri	2.64	1.59	1.51	0.00	9.3	113.58	167
Meerut	0.96	0.96	0.90	0.00	3.72	28.3	41.62
Thiruvallur	1.11	0.87	0.81	0.00	3.85	66.11	97.21

*The denominator used is per 10,000 total population.

** The numerator used is the number of midwives obtained from facility records.

**Fig 2:**
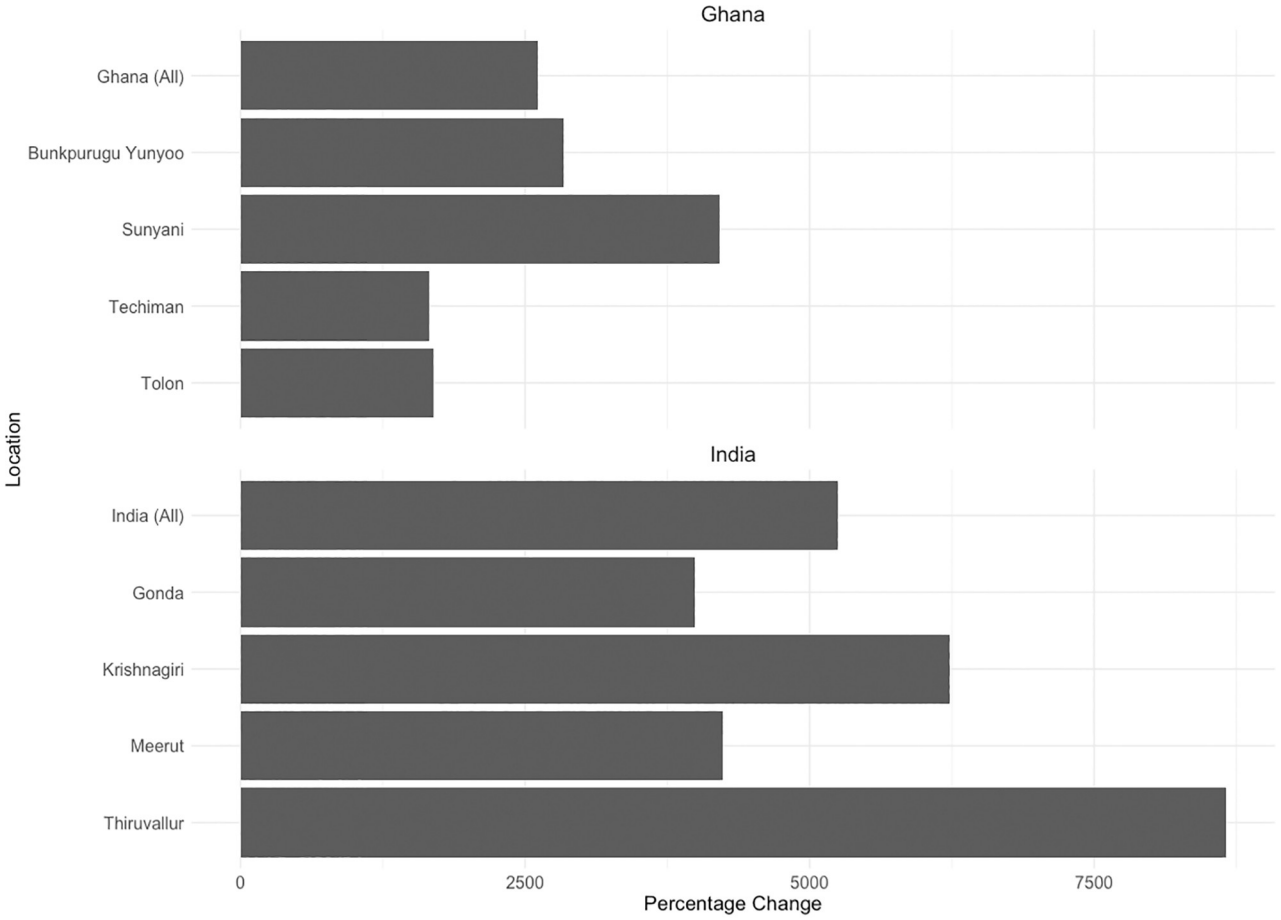
Percentage change in the value of the indicator measuring midwifery density when comparing alternative population denominators.

In redefining the denominator, we observed disproportionate changes in the value of the indicator when comparing estimates obtained per 10,000 total population to per 10,000 births. For example, in Tolon, the value of the indicator increased by approximately 1700% when comparing births instead of total population, while in Thiruvallur, the value of the indicator increased by >8700%. The magnitude of the variation in the change of value of the indicator depending on the population denominator used is shown in Fig 2.

## Discussion

Our study is the first to our knowledge to assess the validity of measures to assess the density and distribution of midwives, which are critical to maternal health system strengthening and performance monitoring. Our results show that varying the denominator used to estimate the population in need of midwifery service coverage vastly changes the value of the estimates for midwifery density. While the most valid (in this context, meaningful) parameter to use for the population in need has not been verified by research, among the plausible options available, it is unlikely to be total population, although this parameter may be the most accurate one available in some contexts.

Our results uncover threats to validity of the numerator that also affect the value of the estimate. With respect to scope of practice, we defined midwives/midwifery professionals inclusively, using ILO ISCO-8, and considered eligible all frontline health workers whose jobs corresponded to the occupation of midwife/midwifery professional or associate midwife/midwifery professional. In India, counting the number of midwives based on data extracted from facility records would overestimate the number of midwives because a significant number were found to be ineligible based on their scope of practice. The overcounting was most pronounced in rural areas and at the primary facility level. These data suggest that relying on facility records to identify the number of midwives may lead to inaccuracies in attempts to measure the density and distribution of midwives and may obscure inequities in the distribution of the workforce. In Ghana, while few midwives were excluded after reviewing their scopes of practice, many health workers from professions that do not meet the ICM definition of a midwife were nevertheless carrying out the occupation per the ILO classification due to workforce shortages. Such health workers from other cadres who are nonetheless functioning as midwives are unlikely to have received the training needed to perform tasks critical to their role, which may be evidenced in the relatively large percentage of midwives in Ghana who reported possessing none of the ICM competencies.

With respect to competency, few studies to date attempt to account for essential skills across the midwifery scope of practice in calculating the density of midwives, and as such to provide a measure of “effective coverage” [35–37]. One of the few reports to model this gap between number of personnel and essential competencies, to our knowledge, was the State of the World’s Midwifery 2014 [13]. Our findings suggest that if the indicator does not account for competency, it may not capture important disparities between urban and rural areas and by facility level, raising important questions of equity. Not accounting for sub-national variability in competency may also mask disparities in the density and distribution of effective coverage.

Targets for density and distribution of midwives to meet the needs of the population have not been set by global stakeholders and thus the “right” or optimal estimate is unknown. As a result, our study can only demonstrate the magnitude of change to the estimate that results from varying the parameters used to calculate it and compare the resulting values to existing or proposed coverage targets. Such existing benchmarks include WHO estimates of 2.5/1000 health workers—including physicians, nurses and midwives—needed to achieve effective coverage of essential primary care interventions, to 4.45/1000 to achieve universal health coverage [16, 49], or midwifery coverage estimates ranging from one to nearly two midwives per 175 births [50, 51]. To ground the indicator in empirical evidence of effective coverage, future research should compare various estimates of midwifery density to health system process measures, such as the percent of women and newborns receiving recommended care, or outcome measures such as rates of mortality, morbidity, or other indicators of health status.

Among this study’s strengths are that it reflects a large, representative sample of midwives and midwifery professionals. Data were examined from eight districts in two countries with varying health system profiles within each setting, thus the results reflect diverse contexts. Further, our expanded definition of midwives and midwifery professionals goes beyond title or designation to account for scope of practice and competency, thus providing a more meaningful estimate of sufficiency beyond the simple number of available midwifery professionals.

At the same time, our findings are subject to some limitations. The data we collected on competency were self-reported. As such, we cannot ascertain the reason for the low number of midwives found to be fully competent. Confounding and bias are possible—for example, this finding could be related to reporting bias (i.e., midwives underreport their skills when asked), which could vary by culture, or could reflect a contextual reason (e.g., related to specific skills not often used due to the organization of care and division of labor, or to changes in clinical practice patterns such that some skills and behaviors are performed less frequently). Factors related to the skills and behaviors emanating from the ICM essential competencies themselves must also be considered, including that the skills and behaviors may not reflect the essential tasks carried out by midwifery professionals in all contexts, or that the complex, composite descriptions of skills and behaviors included in each sub-component for each category of the ICM competencies made it difficult for respondents to assign themselves full competency with precision. These considerations may have practical implications with respect to use of the ICM competencies as measurement tools. They suggest the ICM competencies should be reviewed and revised if all the items included are essential to basic midwifery practice and if they are to be used as a measurement tool.

Further, our data were collected during the global COVID-19 pandemic, when some eligible midwifery professionals were unable to respond due to clinical burden, personal illness, or other caretaking responsibilities, or found it necessary to complete the survey quickly due to constraints on their time. Despite these challenges, our study had very low attrition; less than <1% in both Ghana and India, and very low non-response to survey questions.

Lastly, it is important to note that it is often more difficult to get reliable estimates of births and pregnancies than to get reliable estimates of total population. WorldPop utilizes multiple input data sources and a robust estimation method to produce these estimates, but it is important to remember the fact that these are estimates, not actual counts.

## Conclusion

Our results suggest low convergent validity between two indicators that aim to represent the same construct: the density and distribution of midwives sufficient to meet population needs. The optimal value for this indicator is unknown. Importantly, our study shows that the underlying parameters significantly affect the value of the estimate. For the denominator, we present variance in the value of the estimate using an array of population parameters that are potentially more meaningful than total population. For the numerator, our results show how factoring in a measure of competency impacts the measure of effective coverage of midwifery professionals. The density and distribution indicators represent the construct of adequacy or sufficiency of population coverage of midwives, but in accordance with the more recent conceptualization of “effective coverage” a valid measure needs to reflect more than the simple number of midwifery professionals but also their ability to competently provide the interventions (tasks, skills, and behaviors) that define their scope of practice.

## Supporting information

S1 File (DOCX)

## Abbreviations

EPMM: Ending Preventable Maternal Mortality
GIS: geographic information system
ICM: International Confederation of Midwives
ILO: International Labour Organization
ISCO: International Standard Classification of Occupations
MAP: midwifery associate professional
MNH: maternal newborn health
MP: midwifery professional
SDG: Sustainable Development Goals
WHO: World Health Organization
